# A Study of the Prescription Patterns of Antihypertensive Drugs in Patients Attending the Outpatient Department of Medicine at a Tertiary Care Hospital

**DOI:** 10.7759/cureus.88270

**Published:** 2025-07-18

**Authors:** Pramod D Shankpal, Samiksha M Lohi, Shilpa Karande, Vishal N Bonde

**Affiliations:** 1 Pharmacology, Topiwala National Medical College & Bai Yamunabai Laxman Nair Charitable Hospital, Mumbai, IND; 2 Pharmacology, All India Institute of Medical Sciences, Nagpur, IND; 3 Internal Medicine, Topiwala National Medical College & Bai Yamunabai Laxman Nair Charitable Hospital, Mumbai, IND

**Keywords:** combination therapy, comorbidities, european society of hypertension, indian guidelines on hypertension, international society of hypertension, jnc 8 guidelines

## Abstract

Background

Hypertension is a major global public health concern and a leading risk factor for cardiovascular, cerebrovascular, and renal diseases. Despite the availability of evidence-based guidelines for hypertension management, discrepancies often exist between recommended practices and real-world prescribing patterns. This study aimed to evaluate the prescription pattern of antihypertensive drugs in patients attending the outpatient department (OPD) of medicine at a tertiary care hospital and to assess the adherence of prescribed therapies to the Eighth Joint National Committee (JNC 8) guidelines.

Methods

This was a cross-sectional, single-center, observational study conducted over two years, with data collection spanning six months (April to September 2023) in the OPD of medicine of a tertiary care hospital. A total of 334 patients diagnosed with primary hypertension, meeting the inclusion criteria, were enrolled after obtaining informed consent. Data were collected from printed hospital management information system (HMIS)-generated prescriptions, which included diagnosis, past history, and antihypertensive treatment, along with patient interviews. Data regarding demographics, comorbidities, and antihypertensive prescriptions were collected and analyzed. Prescriptions were also evaluated for adherence to JNC 8 guidelines.

Results

Among 334 patients, 182 (54.49%) were male and 152 (45.51%) were female, with a mean age of 57.2 ± 11.39 years. The majority (57.49%) belonged to the 40-60 years age group. A total of 536 antihypertensive drugs were prescribed, with an average of 1.60 ± 0.65 drugs per prescription. Among the 334 prescriptions analyzed, calcium channel blockers (CCBs) were the most commonly prescribed class (76.95%), followed by angiotensin receptor blockers (ARBs) (60.18%), diuretics (27.25%), beta-blockers (BBs) (18.26%), angiotensin-converting enzyme inhibitors (ACEIs) (5.99%), and alpha-blockers (0.6%). Monotherapy was prescribed in 36.23% of cases, while 63.77% received combination therapy, with two-drug combinations being most common (41.62%). Among monotherapy prescriptions, CCBs were preferred (71.9%), followed by ARBs (20.66%). The most frequent two-drug combination was CCB + ARB (61.87%), while the three-drug combination of CCB + ARB + diuretic was most common (60.66%). In four-drug combinations, CCB + ARB + BB + diuretic was most frequently used (76.93%). Comorbidities were present in 52.69% of patients, with diabetes mellitus (68.18%) being the most prevalent. In diabetic hypertensive patients, CCBs (80.83%) were most commonly prescribed; 82.63% of prescriptions adhered to JNC 8 guidelines.

Conclusion

The study revealed a high prevalence of CCB and ARB use, both as monotherapy and combination therapy, in accordance with national and international guidelines. However, prescribing trends were influenced by factors such as physician preference, comorbidities, and individual clinical judgment. Regular prescription audits and awareness programs focusing on evidence-based guidelines are essential to ensure rational antihypertensive drug use and optimal patient outcomes.

## Introduction

Hypertension is one of the most common chronic medical conditions characterized by a persistent elevation in the arterial pressure [1]. According to the World Health Organization, more than 1.28 billion adults aged between 30 and 79 years worldwide are estimated to have hypertension, with a significant proportion residing in low- and middle-income countries [2]. In India, the burden is equally alarming; among individuals aged between 30 and 79 years, the overall prevalence of hypertension is 31.1% in both sexes, 31.6% in males, and 30.5% in females [3]. According to the Joint National Committee (JNC), hypertension is defined as elevated systolic blood pressure (BP) ≥140 mm Hg or diastolic BP ≥90 mm Hg [4]. Primary hypertension, or essential hypertension, is the term applied to 95% of hypertensive patients, where no single identifiable cause can be determined. Primary hypertension results from a complex interaction between multiple genetic and environmental factors [5, 6]. Secondary hypertension is seen in 5% of patients, and specific causes like primary hyperaldosteronism, renal vascular hypertension, pheochromocytoma, etc., can be identified [5].

Hypertension is associated with increased risk of complications such as stroke, myocardial infarction, atrial fibrillation, heart failure, peripheral vascular disease, and renal diseases [5, 6]. Therefore, it is important that once a diagnosis of hypertension is established, BP should be adequately controlled through lifestyle modification, exercise, regular follow-up, and effective antihypertensive drugs. Antihypertensive drugs are mainly prescribed to reduce the mortality and morbidity caused by hypertension and its complications. Various classes of antihypertensive medications, such as beta-blockers (BBs), angiotensin receptor blockers (ARBs), angiotensin-converting enzyme inhibitors (ACEIs), calcium channel blockers (CCBs), and diuretics, have demonstrated efficacy in reducing hypertension-related complications and are considered as first-line options for initial drug therapy [6, 7].

Several guidelines, such as the JNC [8], the European Society of Hypertension (ESH) [9], Indian guidelines for hypertension [10], international guidelines for hypertension [11], etc., are available as treatment guidelines to reduce practice variability and cost, and also to improve rational pharmacotherapy. The purpose of the Eighth Report of the JNC 8 (JNC 8), released in February 2014, is to provide an evidence-based approach to the prevention and management of hypertension. The available guidelines recommend different goal BP levels and drug treatment options according to the patient’s individual clinical needs [8]. Despite the availability of these guidelines, several studies have reported significant gaps between recommended practices and actual prescribing trends. Despite the availability of these guidelines, several studies have reported significant gaps between recommended guidelines and actual prescribing patterns. For example, Suthar and Dumra found 77.88% adherence to JNC‑8 guidelines [12], Sapkota et al. found non-compliance to JNC‑7 ranging from 7.7% to 45.95% across different clinics in Kathmandu Valley [13], and Romday et al. also observed low guideline adherence [14]. Factors contributing to this discrepancy include clinician preference, drug availability, patient affordability, perceived drug efficacy, and institutional policies.

While the prescription patterns of antihypertensive medications have been extensively studied in various parts of the world, limited data exist from our specific regional and institutional setting. Our study contributes new insights by focusing on the outpatient population of a tertiary care hospital in India, where prescribing decisions may differ due to unique demographic and socioeconomic variables. Assessing the prescription patterns of antihypertensive drugs and their alignment with guideline recommendations is crucial for identifying deviations from rational practice. It also helps in understanding the dynamics of clinical decision-making, resource utilization, and patient-specific considerations that influence therapy selection in outpatient settings. In addition to evaluating prescribing patterns in patients with primary hypertension, the study also examined antihypertensive medication use in individuals with common comorbid conditions such as diabetes mellitus, ischemic heart disease, chronic kidney disease, etc., providing a broader clinical perspective on real-world prescribing practices.

The primary objective of this study was to evaluate the prescription pattern of antihypertensive medications in the outpatient department (OPD) of medicine at Topiwala National Medical College & Bai Yamunabai Laxman Nair Charitable Hospital, a tertiary care hospital in Mumbai, while the secondary objective was to assess physicians’ adherence to the JNC 8 guidelines.

## Materials and methods

This cross-sectional, single-center observational study was conducted over a period of two years, from August 2022 to August 2024, with data collection carried out between April and September 2023. The study was conducted in the OPD of medicine of Topiwala National Medical College & Bai Yamunabai Laxman Nair Charitable Hospital, which is a government, urban, tertiary care teaching hospital, following approval from the Institutional Ethics Committee for Academic Research Projects (ECARP; Ref. No. ECARP/2022/130). Based on the average weekly attendance of 105 patients with primary hypertension in the OPD of medicine, a monthly population of 420 patients was estimated. To represent the six-month study period involving approximately 2,520 patients, a sample size of 334 was calculated using a 95% confidence level and a 5% confidence interval, based on an online sample size calculator [15].

Patients were enrolled if they were over 18 years of age, diagnosed with primary hypertension (with or without comorbidities), and visited the OPD of medicine for treatment. Patients were excluded if they did not provide informed consent, were pregnant, presented with a hypertensive crisis, or had secondary hypertension. A non-probability convenient sampling technique was used to enroll patients who met the inclusion criteria during the study period. Data were collected from printed hospital management information system (HMIS)-generated prescriptions, which included diagnosis, past history, and antihypertensive treatment, along with patient interviews. Demographic information, comorbid conditions, and complete details of antihypertensive prescriptions, including drug name, class, dosage, and frequency, were collected. This information was recorded in a structured case record form (CRF). The interviews were conducted by the co-investigator, who approached patients in the waiting area of the OPD of medicine. Written informed consent was obtained from each participant prior to data collection.

To evaluate adherence to the JNC 8 guidelines, each prescription was reviewed according to the criteria specified in the guideline. According to the JNC 8 recommendations, for the general non-Black adult population (the racial background of participants is presented as recorded in the original source data), including individuals with diabetes, the preferred first-line antihypertensive agents include thiazide-type diuretics, CCBs, ACEIs, and ARBs. If BP goals were not achieved within one month of initiating therapy, treatment could be intensified by either increasing the dose of the initial drug or adding a second agent from the other recommended classes. A third drug from the remaining recommended classes could be introduced if BP remained uncontrolled. However, the simultaneous use of both an ACEI and an ARB was not recommended.

Adherence was assessed by determining whether the prescribed antihypertensive regimen aligned with these recommendations. Prescriptions were classified as non-adherent if monotherapy involved agents outside the recommended four classes, such as BBs or alpha-blockers; if, in combination therapy, the second or third drug belonged to a non-recommended class; if diuretic therapy included non-thiazide agents such as loop or potassium-sparing diuretics; or if both an ACEI and an ARB were prescribed together. Further analysis of adherence was carried out across three domains: age groups, comorbid conditions, and prescription patterns.

Statistical analysis was performed using Microsoft Excel version 2019 (Microsoft Corp., Redmond, WA). Categorical variables were presented as frequencies and percentages. Adherence to JNC 8 guidelines was calculated as the percentage of prescriptions that fulfilled the defined criteria.

## Results

Prescriptions were collected from 334 diagnosed cases of primary hypertension and scrutinized for demographic profile and drug prescription pattern over a period of six months, from April 2023 to September 2023. The results are shared below.

Demographic profile of the patients

Out of a total of 334 cases, 182 (54.49%) were male and 152 (45.51%) were female. The male:female ratio was 1.2:1. The mean age of the study population was 57.2 ± 11.39 years (mean ± SD). The range was from 25 years to 91 years. All patients were divided into three age groups: less than 40 years, 40 to 60 years, and more than 60 years. The most commonly affected age group was 40-60 years (n=192, 57.49%), followed by more than 60 years (129, 38.62%). The maximum number of patients was in the pre-hypertension stage (n=211, 63.17%), followed by stage 1 hypertension (n=71, 21.26%). Comorbidities were found in 176 patients. The most common comorbidity was diabetes mellitus (n=120, 68.18%), followed by dyslipidemia (n=24, 13.64%) (Table 1).

**Table 1:** Demographic details of the patients COPD: chronic obstructive pulmonary disease; HTN: hypertension; N: total number of patients

Age groups (N = 334)	Males		Females			Total		
	No.	%	No.	%		No.	%	
<40 years	6	1.8	7	2.1		13	3.89	
40 – 60 years	100	29.94	92	27.54		192	57.49	
> 60 years	76	22.75	53	15.87		129	38.62	
Total	182	54.49	152	45.51		334	100	
Stages of HTN (N = 334)	Males		Females			Total		
	No.	%	No.	%		No.	%	
Normal	6	1.8	12	3.59		18	5.39	
Pre-HTN	113	33.83	98	29.34		211	63.17	
Stage 1 HTN	40	11.98	31	9.28		71	21.26	
Stage 2 HTN	23	6.98	11	3.29		34	10.18	
Total	182	54.49	152	45.51		334	100	
Comorbidities (N = 176)	Males		Females			Total		
	No.	%	No.		%	No.		%
Diabetes mellitus	58	32.95	62		35.23	120		68.18
Dyslipidemia	17	9.66	7		3.98	24		13.64
Ischemic heart disease	6	3.41	6		3.41	12		6.82
COPD	6	3.41	2		1.14	8		4.55
Chronic kidney disease	4	2.27	3		1.7	7		3.98
Bronchial asthma	2	1.14	3		1.7	5		2.84
Total	93	52.84	83		47.16	176		100

Analysis of the prescription pattern

Pattern of the Number of Drugs Prescribed

We analyzed 334 prescriptions of patients diagnosed with primary hypertension, and the total number of drugs prescribed for hypertension was 536. The average number of antihypertensive drugs prescribed per prescription was 1.60 ± 0.65 (mean ± SD), ranging from one to four drugs per prescription. The median number of drugs prescribed was two. All drugs were given in the form of oral tablets. No drug was given by the injectable route. Out of 536 drugs, 313 (58.4%) were prescribed by generic names and 223 (41.6%) were prescribed by brand names.

Frequency of Administration of Antihypertensive Drugs

Table 2 shows the most frequently prescribed class of antihypertensive drugs. CCBs were the most commonly prescribed class of antihypertensive drugs (n=257, 76.95%), followed by ARBs (n=201, 60.18%), diuretics (n=91, 27.25%), BBs (n=61, 18.26%), ACEIs (n=20, 5.99%), and alpha-blockers (n=2, 0.6%). Among CCBs, amlodipine was most commonly prescribed (n=221, 66.17%). Among ARBs, telmisartan (n=175, 52.39%) was most commonly prescribed (Table 2).

**Table 2:** Frequency of administration of antihypertensive drugs

Antihypertensive class	Name of the drug	Number of prescriptions (N=334)	Percentage
Calcium channel blockers (N=257)	Amlodipine	221	66.17
	Cilnidipine	29	8.68
	Nifedipine	7	2.09
Angiotensin receptor blockers (N=201)	Telmisartan	175	52.39
	Losartan	16	4.79
	Olmesartan	10	2.99
Diuretics (N=91)	Hydrochlorothiazide	37	11.08
	Chlorthalidone	34	10.18
	Furosemide	18	5.39
	Spironolactone	2	0.6
	Indapamide	1	0.3
	Torasemide	1	0.3
Beta-blockers (N=61)	Metoprolol	53	15.87
	Atenolol	7	2.1
	Bisoprolol	1	0.3
Angiotensin-converting enzyme inhibitors (N=20)	Ramipril	14	4.19
	Enalapril	5	1.5
	Perindopril	1	0.3
Alpha-blockers (N=2)	Prazosin	2	0.6

Frequency of Drugs Prescribed as Monotherapy and Combination Therapy

Table 3 shows the number of drugs prescribed in our study as monotherapy and combination therapy. One hundred and twenty-one (36.23%) received monotherapy, and 313 (63.77%) received combination therapy. Out of 334 prescriptions analyzed, the maximum number of prescriptions contained two-drug combinations (n=139; 41.62%), while 61 (18.26%) prescriptions contained three-drug combinations and 13 (3.89%) prescriptions contained four-drug combination therapies.

**Table 3:** Number of drugs prescribed as monotherapy and combination therapy

Number of antihypertensive medications per prescription	Number of prescriptions (N=334)	Percentage
Monotherapy	121	36.23
Two	139	41.62
Three	61	18.26
Four	13	3.89

Prescribing Pattern of Antihypertensive Monotherapy

Prescribing patterns of antihypertensive monotherapy in the treatment of primary hypertension have been depicted in Table 4. Among monotherapy (N=121), CCBs were the most commonly prescribed class (n=87, 71.9%) as monotherapy, followed by ARBs (n=25, 20.66%), BBs (n=5, 4.13%), and ACEIs (n=4, 3.31%).

**Table 4:** Prescribing pattern of antihypertensive monotherapy

Class	Number of prescriptions (N=121)	Percentage
Calcium channel blockers	87	71.9
Angiotensin receptor blockers	25	20.66
Beta-blockers	5	4.13
Angiotensin-converting enzyme inhibitors	4	3.31
Total	121	100

Prescribing Pattern of Antihypertensive Combination Therapy

Table 5 shows the prescribing pattern of antihypertensive two-drug combination therapy in patients with primary hypertension. Nine different combinations of two drugs were prescribed. Amongst these, the combination of CCB + ARB was most commonly prescribed (n=86, 61.87%), followed by the combination of ARB + diuretic (n=23, 16.55%) and CCB + BB (n=12, 8.63%).

**Table 5:** Prescribing pattern of antihypertensive two-drug combination therapy ACEI: angiotensin-converting enzyme inhibitor; ARB: angiotensin receptor blocker; CCB: calcium channel blocker

Two-drug combinations	Number of prescriptions (N=139)	Percentage
CCB + ARB	86	61.87
ARB + diuretic	23	16.55
CCB + Beta-blocker	12	8.63
CCB + ACEI	7	5.03
ARB + Beta-blocker	3	2.16
Beta-blocker + Diuretic	3	2.16
CCB + Diuretic	2	1.44
ACEI + Beta-blocker	2	1.44
ACEI + Diuretic	1	0.72
Total	139	100

Table 6 shows the prescribing pattern of antihypertensive three-drug combinations in the treatment of primary hypertension. Eight different combinations of three drugs were prescribed. Amongst these, the combination of CCB + ARB + diuretic was most commonly prescribed (n = 37; 60.66%), followed by CCB + ARB + BB (n = 8; 13.11%) and ARB + BB + diuretic (n = 7; 11.48%).

**Table 6:** Prescribing pattern of antihypertensive three-drug combination therapy ACEI: angiotensin-converting enzyme inhibitor; ARB: angiotensin receptor blocker; BB: beta-blocker; CCB: calcium channel blocker

Three-drug combinations	Number of prescriptions (N=61)	Percentage
CCB + ARB + Diuretic	37	60.66
CCB + ARB + BB	8	13.11
ARB + BB + Diuretic	7	11.48
CCB + BB + Diuretic	3	4.91
CCB + ACEI + BB	2	3.28
ACEI + BB + Diuretic	2	3.28
CCB + ARB + Alpha-blocker	1	1.64
ARB + BB + Alpha-blocker	1	1.64
TOTAL	61	100

Table 7 shows the prescribing pattern of the antihypertensive four-drug combination in the treatment of primary hypertension. Four different combinations of four drugs were prescribed. Amongst these, the combination of CCB + ARB + BB + diureticwas most commonly prescribed (n = 10; 76.93%), followed by CCB + ACEI + BB + diuretic (n = 1; 7.69%), ACEI + BB + diuretic + diuretic (n = 1; 7.69%), and CCB + BB + diuretic + diuretic (n = 1; 7.69%).

**Table 7:** Prescribing pattern of antihypertensive four-drug combination therapy ACEI: angiotensin-converting enzyme inhibitor; ARB: angiotensin receptor blocker; CCB: calcium-channel blocker

Four-drug combinations	Number of prescriptions (N=13)	Percentage
CCB + ARB + Beta-blocker + Diuretic	10	76.93
CCB + ACEI + Beta-blocker + Diuretic	1	7.69
ACEI + Beta-blocker + Diuretic + Diuretic	1	7.69
CCB + Beta-blocker + Diuretic + Diuretic	1	7.69
TOTAL	13	100

Prescribing Patterns in Various Comorbidities

Table 8 shows the overall prescribing frequency of antihypertensive drugs prescribed either as monotherapy or combination therapy in patients with different comorbidities. Among patients with diabetes mellitus as a comorbidity, CCBs (n=97, 80.83%) were most commonly prescribed, followed by ARBs (n=69, 57.5%).

**Table 8:** Prescribing frequency of antihypertensive drugs in patients with comorbidities ACEI: angiotensin-converting enzyme inhibitor; ARB: angiotensin receptor blocker; CCB: calcium channel blocker; CKD: chronic kidney disease; COPD: chronic obstructive pulmonary disease; IHD: ischemic heart disease; N: total number of patients

Comorbidity	Antihypertensive class											
	CCB		ARB		Beta-blocker		ACEI		Diuretic		Alpha blocker	
	No.	%	No.	%	No.	%	No.	%	No.	%	No.	%
Diabetes mellitus (N=120)	97	80.83	69	57.5	21	17.5	10	8.33	28	23.33	-	-
Dyslipidemia (N=24)	20	83.33	11	45.83	5	20.83	-	-	8	33.33	-	-
IHD (N=12)	7	58.33	7	58.33	7	58.33	2	16.67	7	58.33	-	-
COPD (N=8)	4	50	5	62.5	1	12.5	-	-	2	25	-	-
CKD (N=7)	5	71.43	3	42.86	6	85.71	-	-	5	71.43	-	-
Bronchial asthma (N=5)	5	100	3	60	-	-	-	-	1	20	-	-

Table 9 shows the percentage of comorbid patients on monotherapy and combination therapy. Out of 120 patients with hypertension and diabetes mellitus, 43 (35.83%) were on monotherapy and 77 (64.17%) were on combination therapy. Among 24 patients with dyslipidemia as a comorbidity, 11 (45.83%) received monotherapy and 13 (54.17%) were on combination therapy. Of the 12 patients having ischemic heart disease as a comorbidity, three (25%) were on monotherapy and nine (75%) on combination therapy. In patients with COPD (n = 8), four (50%) were on monotherapy and four (50%) on combination therapy. All seven patients with a comorbidity of chronic kidney disease were on combination therapy. Among five patients with bronchial asthma, two (40%) received monotherapy and three (60%) combination therapy.

**Table 9:** Percentage of comorbid patients on monotherapy and combination therapy CKD: chronic kidney disease; COPD: chronic obstructive pulmonary disease; DM: diabetes mellitus; IHD: ischemic heart disease

Comorbidity	Monotherapy		Two-drug combination		Three-drug combination		Four-drug combination	
	No.	%	No.	%	No.	%	No.	%
Diabetes mellitus (N=120)	43	35.83	55	45.83	15	12.5	7	5.84
Dyslipidemia (N=24)	11	45.83	6	25	7	29.17	-	-
IHD (N=12)	3	25	2	16.67	5	41.66	2	16.67
COPD (N=8)	4	50	4	50	-	-	-	-
CKD (N=7)	-	-	3	42.86	3	42.86	1	14.28
Bronchial asthma (N=5)	2	40	2	40	1	20	-	-

Percentage of Prescriptions Following JNC 8 Guidelines

One of the objectives of our study was to assess physicians’ adherence to JNC 8 guidelines. Table 10 shows the percentage of prescriptions following the JNC 8 guidelines. Out of 334 prescriptions, 276 (82.63%) prescriptions followed the JNC 8 guidelines, and 58 (17.37%) did not follow the JNC 8 guidelines. 

**Table 10:** Percentage of prescriptions following JNC 8 guidelines CKD: chronic kidney disease; COPD: chronic obstructive pulmonary disease; IHD: ischemic heart disease; No.: total number of patients; JNC 8: Eighth Joint National Committee

Age group	Adherence		Non-adherence	
	No.	%	No.	%
<40 years (N=13)	9	69.23	4	30.77
40-60 years (N=192)	158	82.3	34	17.7
>60 years (N=129)	109	84.50	20	15.5
Total (N=334)	276	82.63	58	17.37
Comorbidity	Adherence		Non-adherence	
	No.	%	No.	%
Diabetes mellitus (N=120)	104	86.67	16	13.33
Dyslipidemia (N=24)	19	79.17	7	29.17
IHD (N=11)	5	45.45	6	54.55
COPD (N=8)	6	75	2	62.5
CKD (N=7)	1	14.29	6	28.57
Bronchial asthma (N=5)	5	100	0	0
Total (N=176)	140	79.55	36	20.45
Prescription pattern	Adherence		Non-adherence	
	No.	%	No.	%
Monotherapy (N=121)	116	95.87	5	4.13
Two-drug combination (N=139)	116	83.45	23	16.55
Three-drug combination (N=61)	35	57.4	26	42.6
Four-drug combination (N=13)	9	69.23	4	30.77
Total (N=334)	276	82.63	58	17.37

## Discussion

The slight male predominance observed in our study (Table 1) is consistent with the findings from studies by Jangir et al. and Lamsal et al., where a higher proportion of male patients was reported (55.14% and 55.08%, respectively) compared to female patients (44.2% and 44.85%) [7, 16].

The average age of the study population of our study is comparable to the study conducted by Anuradha et al., in which the mean age was 59.69 ± 9.74 years [17]. Middle-aged and elderly adults constituted the majority of hypertensive patients in our study (Table 1). These findings align with the study conducted by Venkataraman et al., in which the most commonly affected age group was 30-60 years (52.48%) [18]. The age group 40 to 60 years is a critical period for the onset of hypertension because of the beginning of age-related vascular changes and lifestyle factors such as diet and physical inactivity. Beyond 60 years, the prevalence continues to increase due to compounded effects of aging, such as arterial stiffness, presence of comorbidities, and neurohormonal changes [19]. These might be the reasons for hypertension being commonly found in age groups older than 40 years.

In our study, the average number of drugs prescribed was less than that compared to the study done by Jangir et al., in which the average number of antihypertensives per prescription was 2.52 [7]. It is preferable to keep the average number of drugs per prescription as low as possible since higher figures always lead to increased risk of drug interactions, adverse drug reactions, poor medication compliance, and eventually increased cost of prescriptions.

The finding of CCBs being the most commonly prescribed class (Table 2) was similar to the study done by Lamsal et al., in which CCBs accounted for 53.3% of prescriptions [16]. According to JNC 8 guidelines, CCBs are recommended as first-line treatment for hypertension [8]. Physicians might prefer CCBs over other antihypertensive medications because they are highly effective in reducing BP and they have a favorable side effect profile. Among CCBs, amlodipine was most commonly prescribed (Table 2). Similar findings are observed in the study done by Anuradha et al., in which amlodipine (84.34%) was most commonly prescribed amongst CCBs, followed by cilnidipine (15.3%) and nifedipine (0.71%) [17]. Amlodipine has a long duration of action and milder adverse effects, which may explain its preference among physicians [5, 20, 21].

Most patients were on two-drug combination therapy, followed by monotherapy (Table 3). Findings of our study are similar to a study done by Jangir et al., in which 40.86% of total patients received two-drug combination therapy, followed by monotherapy received by 34.85% of patients [7]. The ESH recommends a stepwise approach starting with combination treatment (preferably a two-drug combination) in most hypertensive patients [9]. Combining two antihypertensive drugs from different classes can achieve better BP control. Physicians, based on their own clinical experience, might favor combination therapy for effective and safe BP management. These could be the reasons for the two-drug combination therapy being most common in our study.

Among patients on monotherapy, CCBs were the most commonly prescribed antihypertensive class, with ACEIs being the least prescribed class (Table 4). The findings of our study are similar to a study done by Anuradha et al., in which CCBs (45.1%) were most frequently prescribed, followed by ARBs (39.84%), BBs (11.27%), and ACEIs (3%) [17]. Among 139 patients receiving two-drug combination therapy, CCB + ARB was the most frequently prescribed combination (Table 5). This finding is similar to a study done by Venkataraman et al., where a combination of CCB + ARB (62.1%) was most commonly prescribed, followed by ARB + diuretic and CCB + BB [18]. Out of 61 patients receiving three-drug combination therapy, CCB + ARB + diuretic was most commonly prescribed (Table 6). This finding is similar to the studies done by Lamsal et al. and Narkar et al., in which a three-drug combination of CCB + ARB + diuretic was most commonly prescribed [16, 22]. 

The most frequently prescribed four-drug combination of CCB + ARB + BB + diuretic (Table 7) in our study was consistent with the findings of a study done by Gupta et al. [23]. Each drug class targets a different aspect of BP regulation. ARBs and CCBs primarily affect vascular tone, BBs reduce cardiac output, and diuretics decrease blood volume. This multi-faceted approach ensures more comprehensive BP control [20,21]. According to the JNC 8 guidelines, when three-drug combinations (ACEI/ARB + CCB + diuretic) do not achieve the target BP, a fourth drug may be added. This approach is effective in managing resistant hypertension [8].

Amongst 176 patients with comorbidities, diabetes mellitus was most commonly observed, followed by (Table 8). This is similar to a study done by Narkar et al., where diabetes mellitus (39%) was the most common comorbidity, followed by dyslipidemia (24%) [22]. In patients with coexisting diabetes mellitus, CCBs were the most commonly prescribed class of drugs overall (Table 8). These findings are almost similar to a study done by Narkar et al., where CCBs and ARBs (85.9%) were the most commonly prescribed class with equal frequency (85.9% each) [22]. JNC 8 recommends ACEI/ARB alone or in combination as preferred agents, with the alternative being CCBs [8]. The preference for CCBs observed in our study is further supported by trials such as the Anglo-Scandinavian Cardiac Outcomes Trial (ASCOT) and the Antihypertensive and Lipid Lowering Treatment to Prevent Heart Attack Trial (ALLHAT), which have demonstrated the usefulness of CCBs as monotherapy or in combination with other antihypertensive agents in diabetic patients [24, 25]. In our study, the reason for CCBs being the most commonly prescribed drug as monotherapy and in combination therapy may be because CCBs are well tolerated, effective in lowering BP, and often required to achieve a target value of BP in diabetic patients.

In patients with dyslipidemia as a comorbidity, CCBs were the most commonly prescribed class, followed by ARBs (Table 8). In a study done by Narkar et al., in hypertensive patients with dyslipidemia, CCB (100%) was most commonly prescribed, followed by ARBs (87.5%), diuretics (81.3%), and BBr (25%) [22]. Our study has similar findings. JNC 8 does not offer any guidelines for patients with dyslipidemia. According to the International Society of Hypertension, in patients with lipid disorders, BP should be lowered preferentially with ARBs/ACEIs and CCBs [11]. In our study, the findings are in accordance with these guidelines.

For those diagnosed with both hypertension and ischemic heart disease, CCBs, ARBs, bisoprolol, and diuretics were prescribed with the same frequency (Table 8). In a study done by Narkar et al., CCBs and BBs (100%) were the most commonly prescribed classes in all the patients with ischemic heart disease (n=18), followed by ARBs (61.1%) and diuretics (44.4%) [22]. Our findings are almost similar to this study. JNC 8 offers no guidelines for treating hypertensive patients with ischemic heart disease. According to Indian Guidelines on Hypertension-III, BBs and CCBs are the drugs of first choice in the management of angina in patients with hypertension associated with IHD. BBs have been shown to reduce the risks of reinfarction and cardiovascular death by 25% in patients with myocardial infarction. Amlodipine has been shown to produce subjective and objective improvement in patients with angina [10]. In our study, the findings are consistent with these guidelines.

Among hypertensive patients with COPD, ARBs were the most commonly prescribed class of antihypertensive agent (Table 8). JNC 8 does not give any guidelines for patients with chronic obstructive pulmonary disease (COPD) as a comorbidity. According to the International Society of Hypertension, ARBs and CCBs, and/or diuretics can be prescribed to patients having COPD as a comorbidity, while BBs may be used in selected patients with ischemic heart disease or heart failure. In our study, prescription patterns in COPD patients are according to these guidelines [11]. BBs are not recommended in COPD and asthma, as they are known to exacerbate these conditions [5, 20, 21]. One patient in our study received BBs. This finding is in contrast with the recommendation given by various guidelines. The reason for prescribing it in our hospital setup may be attributed to good control of BP with BBs over the long term. A cardio-selective BB like metoprolol was prescribed. It has less propensity to exacerbate COPD and asthma as compared to non-selective BB [5,20,21].

In hypertensive patients with chronic kidney disease, BBs were the most commonly prescribed class of antihypertensive agent (Table 8). Findings in our study are in contrast to JNC 8 guidelines, which recommend ACEIs or ARBs as the preferred antihypertensive class, with thiazide diuretics and CCBs as alternatives in patients with CKD as a comorbidity [8]. However, BBs have shown an advantage in chronic kidney disease patients, as they have well-established cardioprotective effects. They reduce BP by affecting the dysregulated sympathetic nervous system, which is common in chronic kidney disease patients [26]. Furthermore, the African American Study of Kidney Disease and Hypertension (AASK) study showed lower rates of end-stage renal disease (ESRD) and death in CKD patients treated with BBs [27].

All hypertensive patients with asthma were prescribed CCBs (Table 8). According to Indian Guidelines on Hypertension-III, CCBs such as amlodipine are relatively safe drugs in this group of patients. ACE inhibitors do not increase bronchial reactivity in these patients. It is recommended that if a cough develops, ARBs should be used as an alternative to ACEIs [10]. Findings in our study are in accordance with these guidelines.

The majority of prescriptions were in accordance with JNC 8 guidelines, while a smaller proportion (17.37%) did not follow these recommendations (Table 10). Among these non-adherent prescriptions, the most frequently observed deviations included the use of BBs as monotherapy or in combination regimens, the use of loop diuretics such as furosemide instead of thiazide-type diuretics, and combinations involving non-recommended drug classes such as alpha blockers. These patterns reflect real-world deviations from guideline-based prescribing and may be influenced by physician preferences, comorbid considerations, or drug availability. Adherence was highest among patients aged >60 years, while the lowest adherence was observed in patients under 40 years of age (Table 10). This suggests that physicians may be more vigilant in adhering to guidelines while treating elderly patients, possibly due to their higher cardiovascular risk [19]. In our study, the highest adherence to JNC 8 guidelines was observed in patients with bronchial asthma, followed by those with diabetes mellitus (Table 10). Although JNC 8 does not provide specific recommendations for the management of hypertension in patients with asthma as a comorbidity, the observed adherence in this group likely reflects clinically appropriate prescribing, particularly the avoidance of BBs, which are contraindicated or used with caution in asthma. This suggests that physicians may still align their prescribing with best practices even when not explicitly outlined in the guideline. The high adherence in diabetic patients is directly supported by the JNC 8 guidelines. Conversely, poor adherence was noted in patients with chronic kidney disease (Table 10) despite JNC 8 providing clear recommendations for the use of ACEIs or ARBs in chronic kidney disease. The low adherence in these subgroups could reflect challenges in real-world practice, such as drug intolerance, patient compliance, or physician preference for individualized regimens. Regarding prescription patterns, adherence was highest in monotherapy and two-drug combinations but declined significantly with three-drug combinations (Table 10). Interestingly, it improved again with four-drug combinations, possibly due to a return to structured prescribing in patients with resistant hypertension requiring multiple agents. These findings suggest that prescribing is a composite practice that is influenced by various factors like considering the severity of the disease, existing comorbidity, cost of the drug, evaluating therapy regularly, giving instruction to patients about the intended use, expected outcomes, potential side effects for each prescribed medication, different practicing guidelines, and the clinical experience/judgment of the treating physician.

Strength of the study

Along with prescribing patterns in primary hypertension, we also studied the antihypertensive medication prescribing trend in patients with common comorbidities. The large sample size and six-month data collection period add robustness to the findings. Furthermore, the study included an audit of adherence to JNC 8 guidelines, providing insights not only into what was prescribed but also into whether it aligned with recommended treatment protocols.

Limitations of the study

Because of the cross-sectional design, we were unable to understand long-term changes in prescription patterns and their impact on BP management in patients. The patients with secondary hypertension were excluded from our study (exclusion criteria); hence, it was not possible to evaluate the prescribing pattern of antihypertensive medication in this subset of patients. Because the study was conducted in a tertiary healthcare center in an urban area, the results cannot be generalized to primary and secondary healthcare centers or rural settings. Additionally, as the study was descriptive in nature, advanced statistical analyses and control for potential confounders were not performed, which limits the ability to draw causal inferences.

## Conclusions

Hypertension continues to be a public health problem due to its association with morbidity and mortality. Hypertension is associated with various comorbidities like diabetes mellitus, ischemic heart disease, chronic kidney disease, etc., which impair the health-related quality of life in hypertensive patients and also increase the cost of therapy. A number of national and international guidelines for the management of hypertension have been published, highlighting monotherapy or combination therapy according to the BP levels and associated comorbidity.

In our study, CCBs and ARBs were the most frequently prescribed drug classes, reflecting prescribing practices aligned with clinical guidelines. A higher proportion of patients received combination therapy, particularly those with comorbidities, indicating a local prescribing preference based on practical therapeutic considerations. These kinds of studies, where prescriptions are audited at regular intervals, will help physicians in modifying their prescription trends as per current guidelines on the management of primary hypertension. Hence, regular training should be provided to prescribers on practicing guidelines and the rational use of drugs in patients with primary hypertension. Such training should also incorporate awareness of contextual factors like patient affordability, drug availability, and the integration of updated national and international guidelines into prescribing decisions. This will help the hypertensive patient’s community get a better quality of life.

Future prospective studies evaluating the impact of prescribing patterns on BP control and clinical outcomes would further enhance our understanding and support evidence-based hypertension management.

## References

[1] Iqbal AM, Jamal SF (2025). Essential hypertension. StatPearls [Internet].

[2] (2025). Hypertension. https://www.who.int/news-room/fact-sheets/detail/hypertension.

[3] (2024). Hypertension among adults aged 30-79 years. http://who.int/data/gho/data/indicators/indicator-details/GHO/prevalence-of-hypertension-among-adults-aged-30-79-years.

[4] (2024). The seventh report of the Joint National Committee on prevention, detection, evaluation, and treatment of high blood pressure. Joint National Committee on [Internet.

[5] Kotchen AT (2022). Hypertension. Harrison’s Principles of Internal Medicine.

[6] Sutters M (2022). Systemic hypertension. Current Medical Diagnosis & Treatment.

[7] Jangir RK, Ali A, Ahamed J, Gehlot A, Vyas A, Batar KK (2019). A prospective study of prescription patterns of antihypertensive drugs in hypertensive patients at a tertiary care hospital. J Med Sci Clin Res.

[8] James PA, Oparil S, Carter BL (2025). 2014 evidence-based guideline for the management of high blood pressure in adults: report from the panel members appointed to the Eighth Joint National Committee (JNC 8). JAMA.

[9] Mancia G, Kreutz R, Brunström M (2023). 2023 ESH Guidelines for the management of arterial hypertension The Task Force for the management of arterial hypertension of the European Society of Hypertension: Endorsed by the International Society of Hypertension (ISH) and the European Renal Association (ERA). J Hypertens.

[10] The Association of Physicians of India (2013). Indian guidelines on hypertension - III. J Clin Prev Cardiol.

[11] Unger T, Borghi C, Charchar F (2020). 2020 International Society of Hypertension Global hypertension practice guidelines. Hypertension.

[12] Suthar KM, Dumra GH (2022). Drug utilization study of anti-hypertensive drugs and prescription pattern adherence with joint national committee-8 guideline. Int J Basic Clin Pharmacol.

[13] Sapkota B, Shrestha H, Khatri N, Shreshtha K (2019). Prescribing pattern of anti-hypertensive drugs and adherence to JNC VII guideline. Proceedings.

[14] Romday R, Gupta AK, Bhambani P (2016). An assessment of antihypertensive drug prescription patterns and adherence to joint national committee-8 hypertension treatment guidelines among hypertensive patients attending a tertiary care teaching hospital. Int J Res Med Sci.

[15] (2024). Sample size calculator. https://www.calculator.net/sample-size-calculator.html.

[16] Lamsal KS, Neupane KR, Kafle RS (2020). Prescription patterns of antihypertensive drugs at tertiary care hospital: a descriptive cross sectional study. J Nobel Med Coll.

[17] Anuradha VP, Muhas C, Sruthy SA (2021). Drug utilization pattern of antihypertensive medications in a tertiary care hospital, South India. Asian J Pharm Clin Res.

[18] Venkataraman R, Rashid M, Shashikantha B, Soumya A, Vijayan G, Manuel GG, Islam S (2019). Prescribing pattern of antihypertensive medication and adherence to Joint National Commission-8 guidelines in a rural tertiary care Indian teaching hospital. J Basic Clin Physiol Pharmacol.

[19] Oliveros E, Patel H, Kyung S, Fugar S, Goldberg A, Madan N, Williams KA (2020). Hypertension in older adults: assessment, management, and challenges. Clin Cardiol.

[20] Eschenhagen T (2023). Treatment of hypertension. Goodman & Gilman”s The Pharmacological Basis of Therapeutics, 14th Edition.

[21] Benowitz NL (2024). Antihypertensive agents. Katzung’s Basic & Clinical Pharmacology, 16th Edition.

[22] Narkar NS, Deshpande T, Rane BT, Kothari R, Tilak AV, Bhide H (2025). Pattern of antihypertensive drugs prescribed in a tertiary care hospital in western India. Biomed Pharmacol J.

[23] Gupta SK, Nayak RP, Rahavi R, Kumar A (2015). A cross-sectional retrospective study to assess the pattern of prescribing for inpatient hypertensive cases in a tertiary hospital and to find out the possible avenues for betterment of hypertension management. Arch Med Health Sci.

[24] Ostergren J, Poulter NR, Sever PS (2008). The Anglo-Scandinavian Cardiac Outcomes Trial: blood pressure-lowering limb: effects in patients with type II diabetes. J Hypertens.

[25] (2002). Major outcomes in high-risk hypertensive patients randomized to angiotensin-converting enzyme inhibitor or calcium channel blocker vs diuretic: the Antihypertensive and Lipid-Lowering Treatment to Prevent Heart Attack Trial (ALLHAT). JAMA.

[26] Pugh D, Gallacher PJ, Dhaun N (2019). Management of hypertension in chronic kidney disease. Drugs.

[27] Wright JT Jr, Bakris G, Greene T (2002). Effect of blood pressure lowering and antihypertensive drug class on progression of hypertensive kidney disease: results from the AASK trial. JAMA.

